# Axonal Transport and Local Translation of mRNA in Neurodegenerative Diseases

**DOI:** 10.3389/fnmol.2021.697973

**Published:** 2021-06-14

**Authors:** Seiichi Nagano, Toshiyuki Araki

**Affiliations:** ^1^Department of Neurotherapeutics, Osaka University Graduate School of Medicine, Osaka, Japan; ^2^Department of Peripheral Nervous System Research, National Institute of Neuroscience, National Center of Neurology and Psychiatry, Tokyo, Japan

**Keywords:** axonal transport, local translation, mRNA, amyotrophic lateral sclerosis, frontotemporal dementia, spinal muscular atrophy, Alzheimer’s disease, fragile X syndrome

## Abstract

Since neurons have long neurites including axons, it is crucial for the axons to transport many intracellular substances such as proteins and mitochondria in order to maintain their morphology and function. In addition, mRNAs have also been shown to be transported within axons. RNA-binding proteins form complexes with mRNAs, and regulate transport of the mRNAs to axons, as well as locally translate them into proteins. Local translation of mRNAs actively occurs during the development and damage of neurons, and plays an important role in axon elongation, regeneration, and synapse formation. In recent years, it has been reported that impaired axonal transport and local translation of mRNAs may be involved in the pathogenesis of some neurodegenerative diseases. In this review, we discuss the significance of mRNA axonal transport and their local translation in amyotrophic lateral sclerosis/frontotemporal dementia, spinal muscular atrophy, Alzheimer’s disease, and fragile X syndrome.

## Introduction

Neurodegenerative diseases chronically affect neurons in a specific network of the central or peripheral nervous system. Aging is one of the risk factors for the occurrence of these diseases, and the number of patients is increasing year by year with the increasing elderly population (Heemels, 2016). The detailed etiology of these diseases is still undetermined, and there are currently no curative therapeutics. Therefore, it is extremely important to clarify the pathogenic mechanism of these diseases, and to develop new treatment strategies based on identified mechanisms.

It has been suggested that in some of these neurodegenerative diseases, the function of neurons is impaired due to abnormal aggregation/deposition of the causative proteins inside or outside the neurons. For example, in amyotrophic lateral sclerosis (ALS) and frontotemporal dementia (FTD), changes in the localization of transactive responsive-DNA binding protein 43 (TDP-43) (Arai et al., 2006; Neumann et al., 2006) and fused in sarcoma (FUS) (Kwiatkowski et al., 2009; Vance et al., 2009), as well as formation of inclusion bodies containing these proteins are seen in neurons. It is also known that in some ALS/FTD cases, mRNA and abnormally translated dipeptide repeat protein by repeat-associated non-ATG translation are deposited in neurons due to abnormal expansion of the hexanucleotide repeat sequence in the untranslated region of the *C9orf72* gene (DeJesus-Hernandez et al., 2011; Renton et al., 2011; Mori et al., 2013). Alzheimer’s disease is characterized by the formation of amyloid plaques from extracellular accumulation of amyloid β and neurofibrillary tangles due to aggregation of tau protein in neurons (Serý et al., 2013).

In other neurodegenerative diseases, the pathogenic mechanism is assumed to be due to functional decline or loss of the causative gene. In spinal muscular atrophy (SMA), deletion or missense mutations of the survival of motor neuron 1 (*SMN1*) gene causes loss of functional SMN protein, resulting in damage of motor neurons (Fallini et al., 2012). In fragile X syndrome (FXS), abnormal trinucleotide repeat expansion in the untranslated region of the fragile X mental retardation 1 (*FMR1*) gene reduces fragile X mental retardation protein (FMRP) production, resulting in autism spectrum-like symptoms (Maurin et al., 2014).

In all of these diseases, it is speculated that impaired axonal transport in neurons, especially axonal transport of mRNAs and local translation in axons, are involved in the pathogenesis. Transport and translation of mRNAs in axons are thought to play an important role in maintaining the viability of neurons (Khalil et al., 2018). This review outlines the physiological significance of axonal transport of mRNAs, and explains the pathogenic mechanism of neurodegenerative diseases due to its disruption by providing examples of typical diseases.

## Physiological Role of mRNA Transport in Neuronal Axons

Neurons have neurites consisting of axons and dendrites, which are morphological features characterizing neurons apart from other cells. At the tip of the axon, a growth cone exists during the development of neurons. After maturation, a pre-synapse structure is made to form synapses with other neurons or effector receptors to support to mutual communication. In order to maintain its morphology and function, neurons constantly transport proteins involved in cytoskeleton and synapse formation or essential intracellular substances, such as mitochondria to neurites (Hirokawa et al., 2009). The transport is actively carried out during neurogenesis and regeneration after neuronal injury for axonal elongation/branching and formation of growth cones/synapses. In addition, the transport is also necessary to maintain normal functions in mature neurons.

In recent years, it has been shown that mRNAs are also transported to axons and locally translated onsite into proteins to maintain axonal morphology and function (Holt et al., 2019). Two types of axonal transport are known: fast axonal transport (50–400 mm/day) and slow axonal transport (<8 mm/day). Generally, organelles such as mitochondria are carried by fast axonal transport, whereas cytoskeletal proteins and some soluble proteins are moved via slow transport (Maday et al., 2014). However, even the fast axonal transport is not sufficient for neuronal functions, including synaptic neogenesis or remodeling during long-term potentiation, axonal regeneration after neuronal injury. To enable immediate protein supply at the axon terminal, mRNAs may need to be transported and stored in axons in advance, then locally translated into proteins when necessary.

It is known that factors necessary for protein translation, such as ribosomes, tRNAs, and translation initiation factors are all localized within axons (Kar et al., 2013), and that these translation apparatuses work together to perform local translation. When brain-derived neurotrophic factor (BDNF) or netrin-1, which promotes axon elongation, growth cone and synapse formation, and remodeling, is applied to the axons of cultured neurons, local protein synthesis increases, suggesting that local translation is an important factor in these phenomena (Cagnetta et al., 2018). BDNF binds to its receptor, tropomyosin-related kinase B (TrkB) and activates various intracellular signaling cascades to release mRNAs from RNA granules and promote their local translation (Leal et al., 2014). Deleted in colorectal carcinoma (DCC), a receptor for netrin-1, binds to translation initiation factors and ribosomes directly inside cells, and then promotes local translation in axons by signaling netrin-1 (Tcherkezian et al., 2010).

Ribosomes in axons were originally considered to be transported from cell bodies in neurons or other cells, such as glial cells, after being assembled in the nucleolus (Court et al., 2008). However, it has recently been reported that some ribosomes are assembled in axons in order to maintain their function, from locally translated components such as ribosome proteins as described later (Shigeoka et al., 2019; Nagano et al., 2020). It is thought that neurons have a translation control mechanism specialized for axons, other than the one in cell bodies.

Vesicles, such as endosomes and lysosomes, and mitochondria are crucial to maintain local translation in axons. RNA granules are transferred to axons on lysosomes tethered by annexin A11 (ANXA11), an RNA granule-associated phosphoinositide-binding protein (Liao et al., 2019). Mitochondria are recruited to branching sites of axons following translational machinery to provide energy supply for local translation (Spillane et al., 2013). Furthermore, late endosomes regulate overall protein synthesis in axons by association with both RNA granules and ribosomes, and by controlling mitochondrial function through translation of the related mRNAs (Cioni et al., 2019).

## Significance of Axonal Degeneration in Neurodegenerative Diseases

It is controversial which is more critical for triggering neuronal cell death in neurodegenerative diseases: changes in neuronal cell bodies (neuronopathy) or changes in axons (axonopathy). Nonetheless, there are many reports that indicate changes in axons or synapses occur during the early stage of the diseases. In ALS and SMA, morphological and functional abnormalities of the neuromuscular junctions between lower motor neurons and skeletal muscles, are detected in model mice that have not yet developed motor symptoms, as well as in patients with mild symptoms (Fallini et al., 2012; Moloney et al., 2014). In Alzheimer’s disease (AD), abnormally aggregated Aβ oligomers inside and outside of neurons have been shown to suppress long-term potentiation at synapses or alter the synaptic structure (Viola and Klein, 2015). In addition, changes suggestive of impaired axonal transport, such as axonal swelling and tau deposition in axons, have been observed in pre-symptomatic model mice and early symptomatic patients (Stokin et al., 2005).

## mRNA Transport and Local Translation in Axons in Neurodegenerative Diseases

As mentioned above, local translation in axons plays an important role in maintaining a healthy state of neurons. Therefore, it is presumed that if the local translation is disrupted, the function and viability of neurons will be impaired. In fact, several neurodegenerative diseases have been suggested to impair the local translation function, and are considered to be involved in the pathophysiology of various neurodegenerative disease models. In the following, we review dysfunctions of local translation in some typical neurodegenerative diseases.

### Amyotrophic Lateral Sclerosis/Frontotemporal Dementia

Amyotrophic lateral sclerosis is an intractable disease in which impairment of upper and lower motor neurons cause muscle weakness and atrophy of skeletal muscles throughout the body. FTD is accompanied with personality changes, behavioral abnormalities, aphasia, etc., due to degeneration of cerebral cortical neurons in the frontal and temporal lobes. Mutations of causative genes, especially those of RNA-binding proteins, have been identified in some cases of these diseases, including TDP-43 (*TARDBP*), *FUS*, heterogeneous nuclear ribonucleoprotein A1 (*hnRNPA1*), *hnRNPA2/B1*. As a frequent mutation in ALS/FTD, abnormal expansion of the GGGGCC repeat sequence in the untranslated region of the *C9orf72* gene is also observed (Mejzini et al., 2019).

In addition to the cases caused by these gene mutations, many sporadic ALS/FTD cases of unknown genetic factors show TDP-43 disappearance from the nuclei and abnormal depositions in the cytoplasm of neurons, indicating that these diseases form a common pathological spectrum in terms of TDP-43 pathology (Arai et al., 2006; Neumann et al., 2006). Similar localization abnormalities are also observed in other RNA-binding proteins such as FUS (Kwiatkowski et al., 2009; Vance et al., 2009). These phenomena imply that disturbance of RNA metabolism due to functional abnormality of RNA-binding proteins is involved in the pathogenesis of ALS/FTD.

Physiologically, TDP-43 and FUS are mainly localized in the nucleus and control gene translation and splicing of transcribed pre-mRNAs. In addition, these proteins shuttle between the nucleus and cytoplasm to regulate export out of the nucleus, transfer into the cytoplasm, and translation of mature mRNAs (Ederle and Dormann, 2017).

mRNAs can form granular structures in the cytoplasm called RNA granules in association with RNA-binding proteins (Khalil et al., 2018). mRNAs are transferred to a required site in RNA granules where translation is suppressed, and when protein synthesis is needed, they are released from the RNA granules and incorporated into translation machinery, such as ribosomes. RNA granules consist of: stress granules that temporarily suppress the translation of mRNAs during stress such as starvation, P-bodies that work to degrade mRNAs, and neuronal RNA granules that transport mRNAs to neurites such as axons and dendrites (Anderson and Kedersha, 2009). These RNA granules contain common RNA-binding proteins and are supposed to have similar structures to each other. A single RNA granule includes multiple RNA-binding proteins to incorporate specific mRNA having an affinity for these proteins.

Transactive responsive-DNA binding protein 43 and FUS are constituents of stress granules, which regulate the translation of mRNAs (Aulas and Vande Velde, 2015). In addition, these proteins also form neuronal RNA granules and are involved in the transport of mRNAs to axons (Alami et al., 2014; López-Erauskin et al., 2018). Both TDP-43 and FUS have a highly hydrophobic amino acid sequence region called low complexity domain (LCD), and multiple molecules of RNA-binding proteins associate with this region to form a non-membranous structure called a liquid droplet. It generates surfaced vesicles and contributes to the formation of RNA granules. Most of the *TARDBP* and *FUS* gene mutations found in familial ALS/FTD are distributed in the LCD of TDP-43, and in the nuclear localization signal (NLS) site required for nuclear localization of FUS. These mutations are thought to be involved in diseases by changing the intracellular localization and enhancing aggregation of the proteins. Mutant TDP-43 and FUS show disturbance of stress granule dynamics (Aulas and Vande Velde, 2015). In addition, neuronal RNA granules containing TDP-43 dynamically change in morphology and assembly in axons, and the number of mature granules decreases by inhibition of hydrophobic binding (Gopal et al., 2017). It has also been reported that in mutant TDP-43 the neuronal RNA granules formed are unstable, and anterograde axonal transport is reduced (Alami et al., 2014).

Previous reports have identified some molecules to be TDP-43-dependent for axonal transport. For instance, neurofilament-L (*NEFL*) and microtubule associated protein 1B (*MAP1B*) mRNAs are reported to be transported to axons by TDP-43 (Godena et al., 2011; Alami et al., 2014). *Drosophila* lacking the TDP-43 gene show abnormal synaptic structure due to a decrease in MAP1B protein at synapses (Coyne et al., 2014). FUS transports *FOSB* mRNA in axons, dysregulation of which causes abnormal axonal branching (Akiyama et al., 2019). It has also been shown that TDP-43 and FUS transport mRNAs to neurites by binding to a three-dimensional structure called G-quadruplex (Ishiguro et al., 2016; Imperatore et al., 2020). In order to survey target mRNAs for axonal transport by TDP-43, we screened mRNAs downregulated in axons by RNAi-mediated TDP-43 down-regulation, and identified mRNAs of translation-related factors including multiple cytoplasmic ribosomal proteins and some translation elongation factors as a major targets of TDP-43-dependent axonal transport (Nagano et al., 2020).

Ribosomal proteins compose ribosomes, which are intracellular apparatuses that together with ribosomal RNA translate proteins from mRNAs. mRNAs of translation-related proteins, including different subunits of ribosomal proteins, are abundant in neuronal axons (Bigler et al., 2017; Rotem et al., 2017), suggesting that the axonal transport of the mRNAs has functional significance. Ribosomal protein mRNAs have been shown to be translated in axons and to play an important role in axonal ribosome assembly (Shigeoka et al., 2019; Nagano et al., 2020). From the results above, it is conceivable that the impairment of axonal protein translation by decreased axonal transport of ribosomal protein mRNAs may lead to the degeneration of motor neurons in ALD/FTD. Furthermore, we have found that the RNA-binding protein La, which is a known antigen of autoantibodies detected in systemic lupus erythematosus and Sjögren’s syndrome (Maraia et al., 2017), is co-localized with TDP-43 and ribosomal protein mRNA in axons. A detailed analysis of the regulatory mechanism of local translation by TDP-43 and La is anticipated.

Recent reports have shown findings similar to our study. Ribosomal protein mRNAs have been identified as mRNAs with decreased stability in fibroblasts and iPS cell-derived motor neurons in sporadic and *C9orf72* mutated ALS patients (Tank et al., 2018). Briese et al. (2020) showed that mRNAs of translation-related proteins including ribosomal proteins are identified as targets for TDP-43-dependent axonal transport in motor neurons, and TDP-43 regulates their local translation. Other reports also showed that mice overexpressing TDP-43 localized in the cytoplasm or mutant FUS have a decrease in overall protein translation in neurons including axons, postulating their involvement in the pathogenesis of ALS/FTD (López-Erauskin et al., 2018; Charif et al., 2020).

Approximately half of FTD cases do not show TDP-43 pathology, but instead such cases tau shows abnormal phosphorylation (Šimić et al., 2016). Also, mutations in the tau (*MAPT*) gene have been identified in juvenile FTD (Hutton et al., 1998). Tau binds to cytoskeletal protein microtubules and contributes to the morphology maintenance of neurons including axons. Abnormally phosphorylated tau undergoes structural changes that reduce its binding to microtubules, and form aggregates and deposits in somatodendritic or axonal compartments (Combs et al., 2019). Microtubules serve as tracks for axonal transport via motor proteins such as kinesin. Therefore, in tauopathy, it is possible that axonal transport of various molecules, including mRNAs, is impaired, which likely disrupts local translation.

### Spinal Muscular Atrophy

Spinal muscular atrophy is a hereditary disorder that causes degeneration of lower motor neurons (Bharucha-Goebel and Kaufmann, 2017). It is classified into types I thru IV according to the age of onset; and the earlier the onset is, the more serious motor symptoms manifest. Pathologically, a decrease in motor neuron axons and morphological changes in the neuromuscular junction are observed at an early stage (Ling et al., 2012; Courtney et al., 2019), and axonal degeneration plays a central role in disease progression. In most SMA cases, deletion of the *SMN1* gene destabilizes its translation product SMN, causing a deficiency in SMN protein expression (Fallini et al., 2012). *SMN2* gene is a paralog of *SMN1* gene, and *SMN2* differs from *SMN1* by only five nucleotides at the 3′ end of the gene. Most *SMN2* transcripts are spliced to skip exon 7 due to a single nucleotide change from *SMN1* in the exon. By that, only about 10% are the full-length transcript to produce a stable protein. The copy number of the *SMN2* gene varies between patients. Most severe type I patients have only 1 or 2 copies of *SMN2*; and the symptoms become milder with an increase of *SMN2* copy number because the *SMN2* gene transcript can help supplement the deficiency of SMN protein to some extent (Calucho et al., 2018).

Survival of motor neuron is ubiquitously expressed throughout the body, but SMA has a particularly profound effect on lower motor neurons. SMN is mostly located in the nucleus and together with gem-associated proteins (Gemins) assembles small nuclear ribonucleoproteins (snRNP) (Fallini et al., 2012) and forms a complex called spliceosome, which executes pre-mRNA splicing (Pellizzoni, 2007). SMN is also localized in axons (Todd et al., 2010) and regulates axonal transport of mRNAs, as well as their translation by interacting with cytoskeletal proteins and RNA-binding proteins (Fallini et al., 2012). SMN binds to actin-binding proteins profilins, and modulates actin polymerization (Giesemann et al., 1999; Sharma et al., 2005). SMN in axons forms a ribonucleoprotein complex with components different from snRNP, suggesting that SMN have a function independent of splicing regulation in snRNP (Fallini et al., 2011). HuD, the neuron-specific RNA-binding protein assembled into RNA granules, controls axonal transport of mRNAs and axon elongation in cooperation with SMN (Akten et al., 2011; Hao le et al., 2017). In addition, it has been reported that axons have a short isoform different from the canonical SMN which acts on polarity formation in neurons (Pletto et al., 2018). The dysfunction of the molecules may define specific changes in motor neurons due to *SMN1* expression deficiency.

Localization of β-actin (*ACTB*) mRNA is reduced in axons of SMN-deficient motor neurons (Rossoll et al., 2003). β-actin is a major skeletal protein that acts on morphological maintenance and dynamics of growth cones and synapses. In addition to axonal targeting of *ACTB* mRNA, SMN also regulates its local translation in growth cones, which is known to be impaired in SMN-deficient motor neurons (Rossoll et al., 2003; Rathod et al., 2012).

Growth-associated protein 43 (*GAP43*) mRNA is also transported and translated in axons under the control of SMN (Fallini et al., 2016). *GAP43* is highly expressed in growth cones and binds to phospholipids in the cell membrane. This protein signals to maintain cytoskeletal morphology (Hartl and Schneider, 2019). Axonal transport of *GAP43* and *ACTB* mRNAs influence each other, and their local translation affects axon elongation and branching, respectively (Donnelly et al., 2013).

Candidate plasticity-related gene 15 (*CPG15*) is highly expressed in developing spinal motor neurons, and promotes axon branching and neuromuscular junction formation. *CPG15* mRNA is transported to axons with the assistance of SMN and translated locally. Overexpression of *CPG15* mRNA rescues SMN deficiency motor axon defects, indicating that localization of *CPG15* mRNA in axons is an important determinant of axon architecture (Akten et al., 2011).

There are other RNA-binding proteins as well, which function in conjunction with SMN. Heterogeneous nuclear ribonucleoprotein R (hnRNP R) binds to both *ACTB* mRNA and SMN (Glinka et al., 2010). Furthermore, HuD is involved in axonal transport of *GAP43* and *CPG15* mRNAs (Akten et al., 2011; Hao le et al., 2017). These RNA-binding proteins are thought to regulate the axonal transport and translation of target mRNAs in cooperation with SMN.

Decreased expression of SMN has been shown to result in reduced overall translation in growth cones (Fallini et al., 2016). This may suggest that the axonal transport and local translation of mRNA regulated by SMN are involved not only in the above-mentioned mRNAs, but also in the metabolic regulation of many mRNAs, or that mRNAs that directly affect the translation function.

### Other Neurodegenerative Diseases

Alzheimer’s disease causes memory deficits, behavioral changes, and personality changes due to cognitive impairment, and is the most common causative disease of dementia. Pathologically, amyloid β (Aβ) is produced by processing from amyloid precursor protein (APP) and extracellularly deposited as amyloid plaques. These plaques and hyperphosphorylated tau which is characterized by intracellular accumulation as neurofibrillary tangles are hallmarks of the disease (Serý et al., 2013). One of the Aβ isoforms, Aβ_1__–__42_, has the most potent aggregating property. Mutations in *APP*, presenilin 1 (*PSEN1*) and *PSEN2* genes have been identified in familial AD patients, and these mutations are responsible for increased processing of Aβ_1__–__42_ (Selkoe and Hardy, 2016).

Brain-derived neurotrophic factor mRNA and BDNF protein are reduced in the brains of AD patients and model mice (Tanila, 2017). *In vitro*, Aβ reduces BDNF signaling by inhibiting the proteolytic production of BDNF from pro-BDNF (Zheng et al., 2010), and by suppressing the retrograde axonal transport of the BDNF-TrkB complex (Poon et al., 2013).

On the other hand, local treatment of Aβ_1__–__42_ on axons has been shown to increase translation from some of the mRNAs within axons (Baleriola et al., 2014). Among them, the transcription factor *ATF4* mRNA is locally translated in axons, and then ATF4 protein is retrogradely transported into the nucleus to increase the expression of C/EBP homologous protein (CHOP), a key molecule of the unfolded protein response pathway, thereby causing neurodegeneration. *ATF4* mRNA and protein are both increased in axons of brain lesions in patients with AD, supporting the notion obtained from cultured neuron experiments.

Furthermore, tau binds to TIA1, one of the RNA-binding proteins in stress granules, and in doing so controls the formation of stress granules and regulates protein translation. Conversely, TIA1 promotes tau aggregation and neurotoxicity (Vanderweyde et al., 2016). In addition, increased expression of G3BP1 and IMP1, which are RNA-binding proteins that bind to *MAPT* mRNA, change the isoform expression pattern of *MAPT* mRNA and protein, increase the formation of neuronal RNA granules, and promote axonal elongation (Moschner et al., 2014).

It is also known that TDP-43 pathology exists in 20–50% of AD patients (Chang et al., 2016). Although it is not yet clear how this change is involved in the pathogenesis of AD, it is quite possible that the dysfunction of TDP-43 is modifying the AD pathology by impairing local translation in neurons with a mechanism similar to ALS/FTD disease models.

Fragile X syndrome is a disease that causes various physical abnormalities, intellectual disability, and psychiatric symptoms. Abnormal expansion of the CGG repeat sequence in the 5′ untranslated region of the *FMR1* gene causes methylation of the promoter of the gene, which decreases the expression of the FMRP at the transcription level for emergence of the disease (Hagerman et al., 2017).

Fragile X mental retardation protein is an RNA-binding protein having a ribonucleoprotein K homology (KH) domain and an arginine–glycine–glycine repeat (RGG) domain, which function to suppress the overall translation of proteins via binding to the RGG domain to G-quadruplexes of mRNAs (Schaeffer et al., 2001), or binding to ribosomes between large and small subunits (Chen et al., 2014).

The mechanism of translation suppression by FMRP has been vigorously investigated primarily in dendrites, specifically in postsynaptic spines (Thelen and Kye, 2020; Lu et al., 2021). However, FMRP is also present in axons and forms a complex with molecules involved in RNA interference (RNAi) such as Dicer, and suppresses the expression of *RHOA* mRNA in distal axons, which causes collapse of growth cones (Hengst et al., 2006). It has also been shown that FMRP transports *MAP1B* and calmodulin (*CALM1*) mRNA into axons, and regulates their local translation via microRNA to act on axon elongation (Wang et al., 2015).

### General View

**FIGURE 1:**
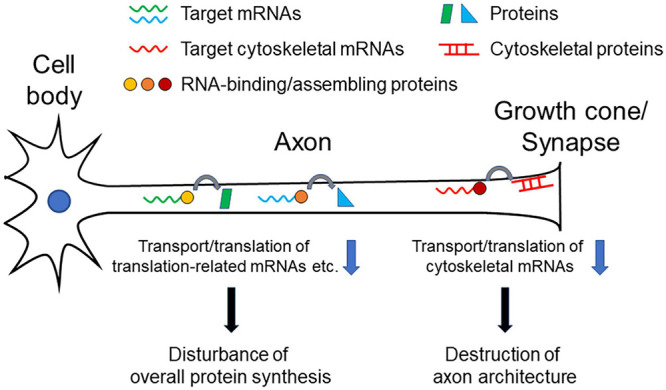
Pathogenetic model of neurodegenerative diseases. Decrease in transport/translation of cytoskeleton-related mRNAs results in inhibiting maintenance of structural integrity of axons, especially of growth cone or synapses, leading neuronal dysfunction and degeneration. Decrease of transport/translation of translation-related (e.g., ribosomal protein mRNAs) mRNAs or many target mRNAs causes dysregulation of overall protein synthesis in axons, which leads neuronal dysfunction and degeneration.

As we overviewed here, different RNA-binding or RNA-assembly protein abnormalities are reported to be associated with neurodegenerative disorders, and the corresponding target mRNAs have been identified in each of the proteins. Notably, many of the target mRNAs are cytoskeleton-related protein transcripts that reside in growth cones and synapses. These data suggest that axonal transport and local translation of these mRNAs are important for the morphological and functional formation and maintenance of growth cones and synapses. Dysfunction of the transport and translation of these mRNAs causes degeneration of axon terminals, which disconnects the neural network to cause symptoms of each disease (Figure 1). Impairment of such functions may also gradually affect neuronal viability, resulting in chronically progressive neurodegeneration. Furthermore, *TUBA4A* and *NEFH* are listed as the causative genes of some ALS cases (Al-Chalabi et al., 1999; Smith et al., 2014), which also indicates the importance of cytoskeletal proteins in the pathogenesis of the disease.

The integrity of ribosomes in axons to maintain local protein synthesis capability may also be a critical factor for maintaining neuronal health. As we discussed above, RNA-binding and RNA-assembling proteins can regulate transport and translation of many different mRNAs in axons, rather than limited specific targets. In addition to our finding that TDP-43 targets translation-related mRNAs for axonal transport, change of overall translation efficiency is detected in axons as a result of TDP-43, FUS, and SMN dysfunction (Rossoll et al., 2003; López-Erauskin et al., 2018; Nagano et al., 2020). Protein synthesizing activity in axons and synapses is indispensable not only for rapid neuronal responses, but also for maintaining structural integrity and electrical conductivity of axons (Figure 1).

## Conclusion

Axonal transport and local translation of mRNAs play an important role in maintaining the survival of neurons, and their failure to do so is greatly involved in the development of many neurodegenerative diseases. By improving the function of mRNA metabolism in axons, it will be possible to develop new therapeutic strategies for these neurodegenerative diseases.

## Author Contributions

SN and TA wrote the manuscript. Both authors contributed to the article and approved the submitted version.

## Conflict of Interest

The authors declare that the research was conducted in the absence of any commercial or financial relationships that could be construed as a potential conflict of interest.
